# Supplementary material for: Biogenesis of DNA-carrying extracellular vesicles by the dominant human gut methanogenic archaeon
Source: Nat Commun. 2025 Jun 3;16:5093. doi: 10.1038/s41467-025-60272-9 (PMC12134362; doi:10.1038/s41467-025-60272-9)
Supplement: Supplementary file 1 — Supplementary Information [file 41467_2025_60272_MOESM1_ESM.pdf]

SUPPLEMENTARY INFORMATION

Biogenesis of DNA-carrying extracellular vesicles by the dominant human gut methanogenic archaeon

Diana P. Baquero[1]*, Guillaume Borrel[2], Anastasia Gazi[3], Camille Martin-Gallausiaux[2], Virginija Cvirkaite-Krupovic[1], Pierre-Henri Commere[4], Nika Pende[2,5], Stéphane Tachon[6], Anna Sartori-Rupp[6], Thibaut Douché[7], Mariette Matondo[7], Simonetta Gribaldo[2], Mart Krupovic[1]*

[1] Institut Pasteur, Université Paris Cité, CNRS UMR6047, Archaeal Virology Unit, 75015 Paris, France
[2] Institut Pasteur, Université Paris Cité, Evolutionary Biology of the Microbial Cell, Paris, France
[3]Institut Pasteur, Ultrastructural Bio Imaging, UTechS, Université Paris Cité, Paris, France
[4] Institut Pasteur, Flow Cytometry Platform, Paris, France.
[5] University of Vienna, Archaea Physiology and Biotechnology Group, 1030 Vienna, Austria
[6] Institut Pasteur, NanoImaging Core Facility, Centre de Ressources et Recherches Technologiques (C2RT), Paris, France
[7] Institut Pasteur, Université Paris Cité, CNRS UAR2024, Proteomics Platform, Mass Spectrometry for Biology, Paris, France

*Correspondence to:
Diana P. Baquero, E-mail: dp.baquero645@gmail.com
Mart Krupovic, E-mail: mart.krupovic@pasteur.fr

Supplementary figures

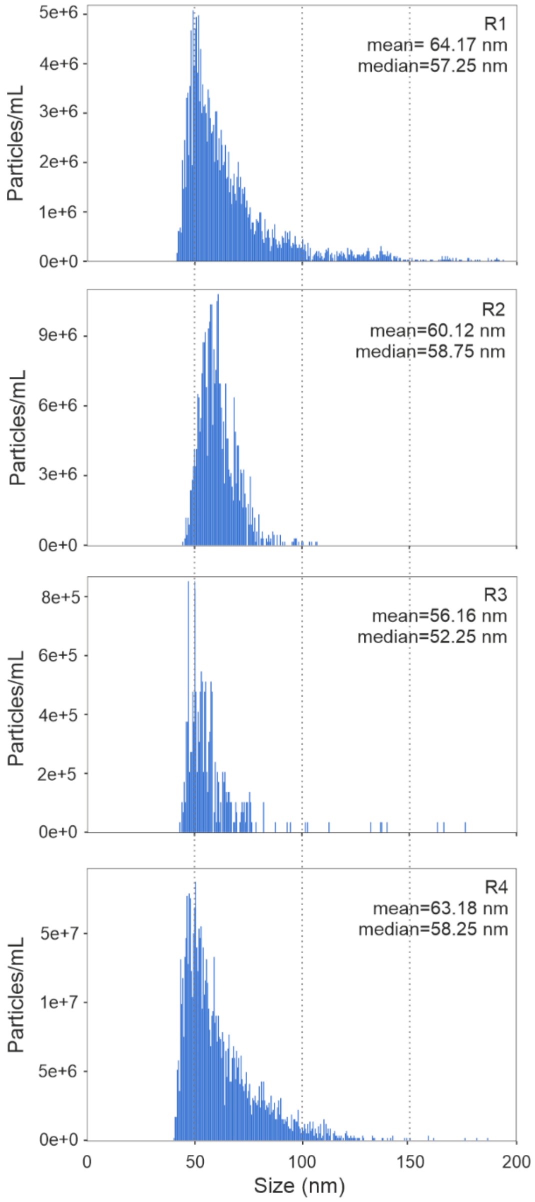

Figure S1. Biological replicates of EV analysis using nanoFCM. Each replicate (R) represents independently purified EV preparations (n=4 independent experiments). Source data are provided as a Source Data file.

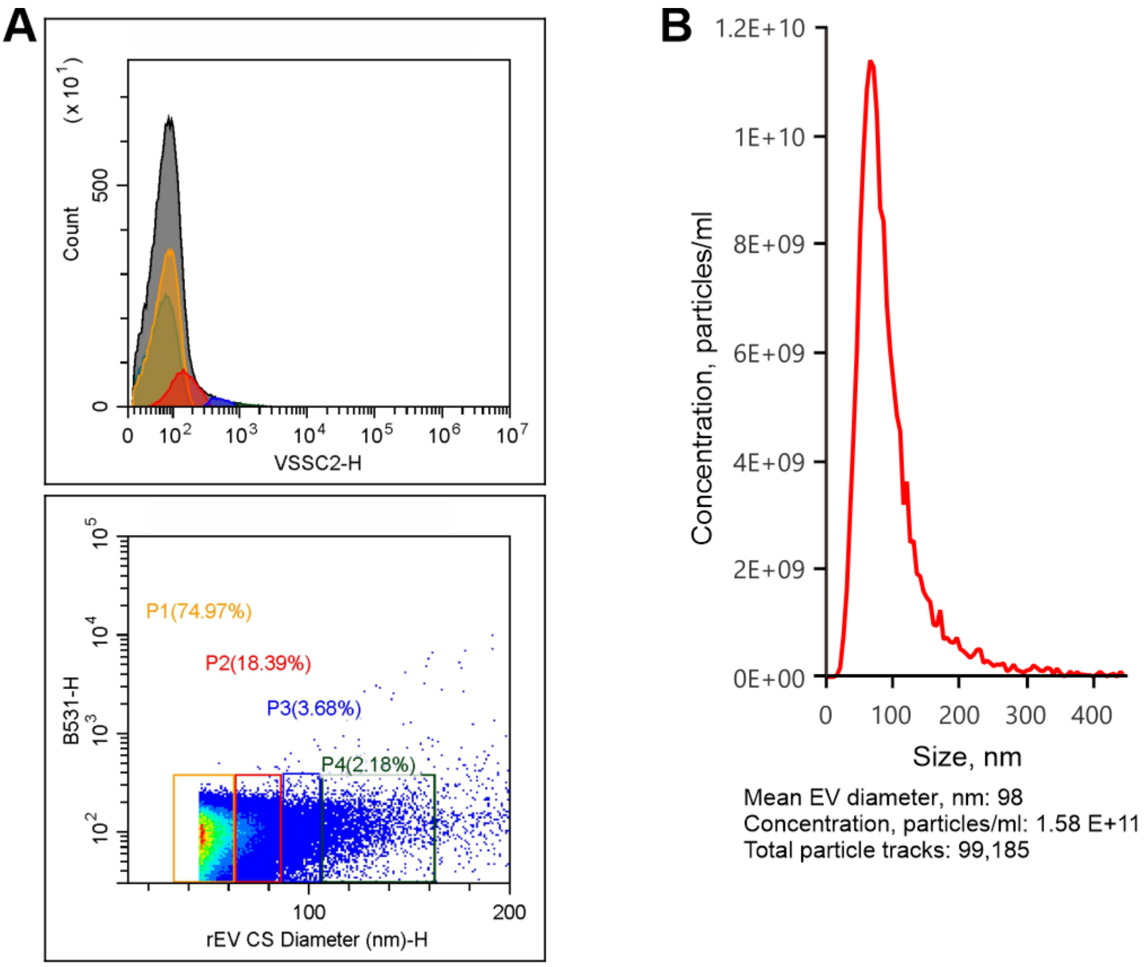

Population	Median EV Diameter (nm)
P1	54.3
P2	78.0
P3	108.9
P4	146.5

Figure S2. Size distribution of *M. smithii* EVs determined using flow cytometer (CytoFLEX Nano, Beckman Coulter) (**A**) and Nanoparticle Tracking Analysis (NanoSight Pro, Malvern Panalytical) (**B**) instruments. The same EV preparation was used in both experiments.

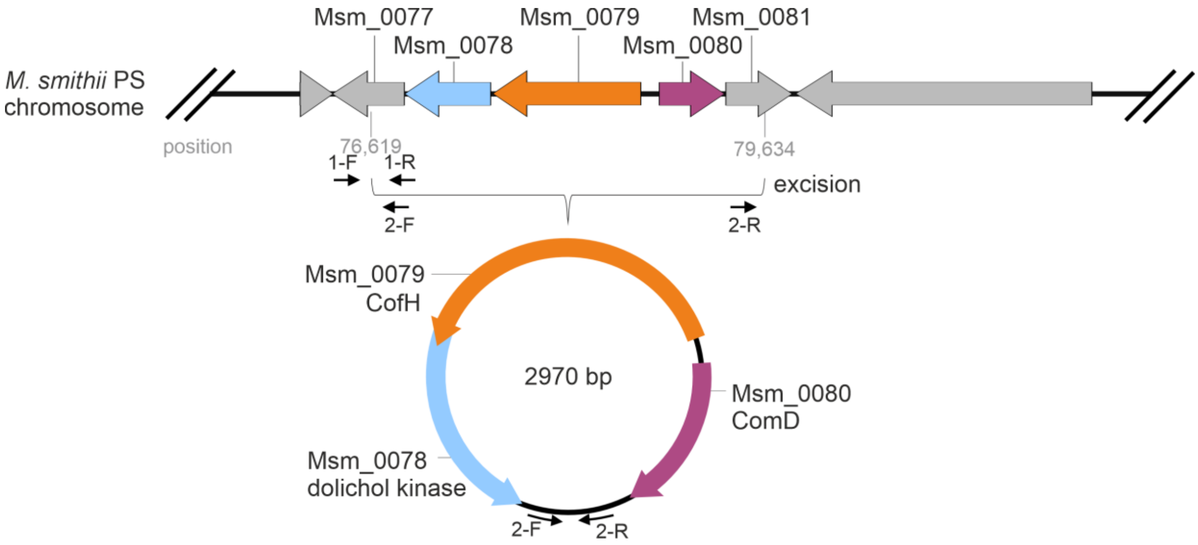

Figure S3. Schematic representation of the excision of the 2970 bp circular element. Two sets of primers (indicated with small black arrows) were designed to detect the integrated and excised forms of the virus genome. The primers 1-F and 1-R targets the sequence integrated into the host chromosome (1-F 5' TCTTCAGGACTTACATCCAGG, R 5' TGTACGTTCACATCCGTCTA). Primers 2-F and 2-R detects the circularized form of the extrachromosomal element (2-F: 5'-CTGTTGAAGAAGGTAAACCCG-3', 2-R: 5'-TTGTACGTTCACATCCGTCT-3'). The PCR results are shown in **Fig. 2C**.

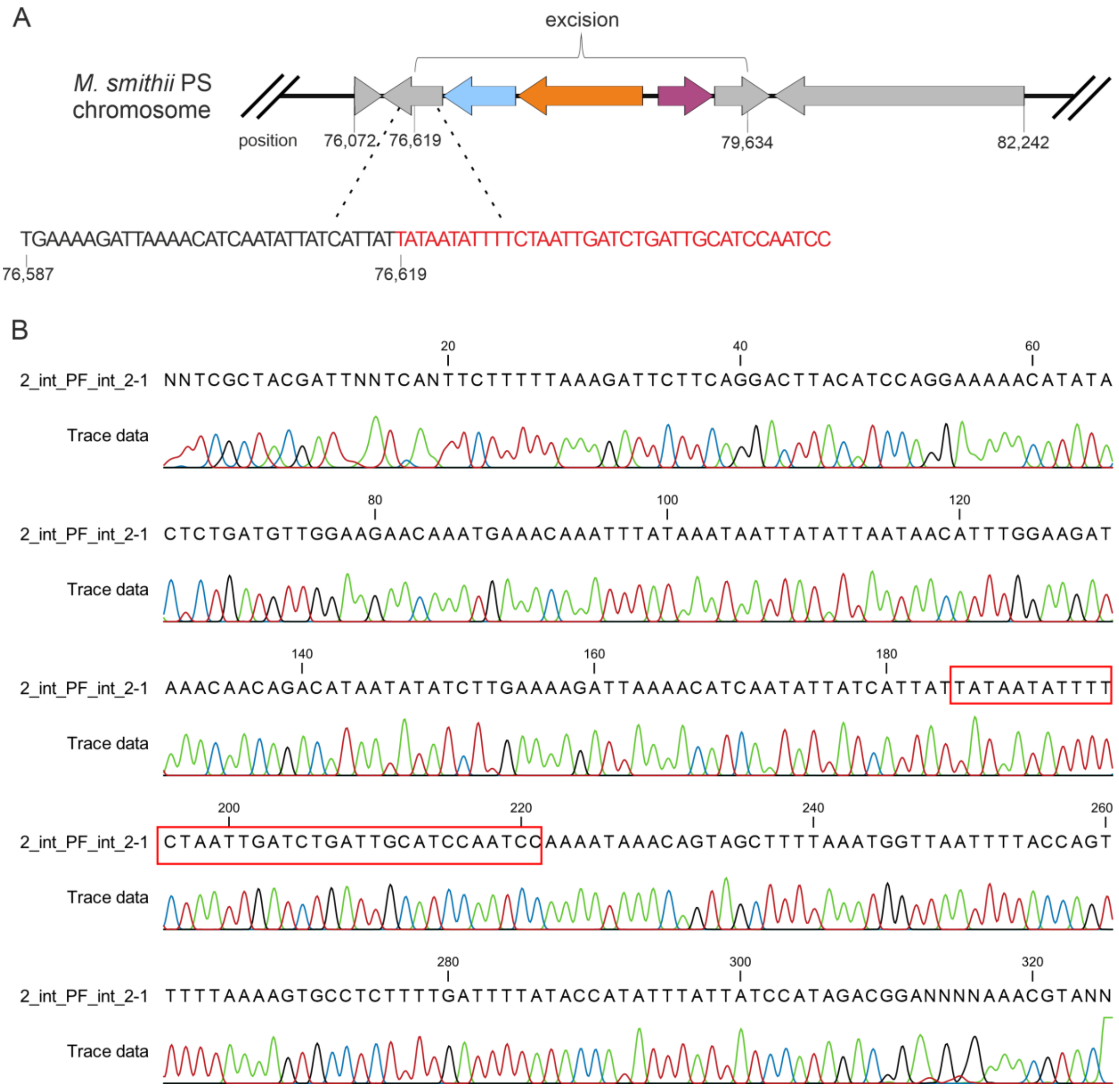

Figure S4. Detection of the element integrated into the host chromosome. **A**, Schematic representation showing the sequence of the circular element integrated into the host genome. **B**, Sequencing chromatogram confirming the results obtained by PCR (**Fig. 2C**).

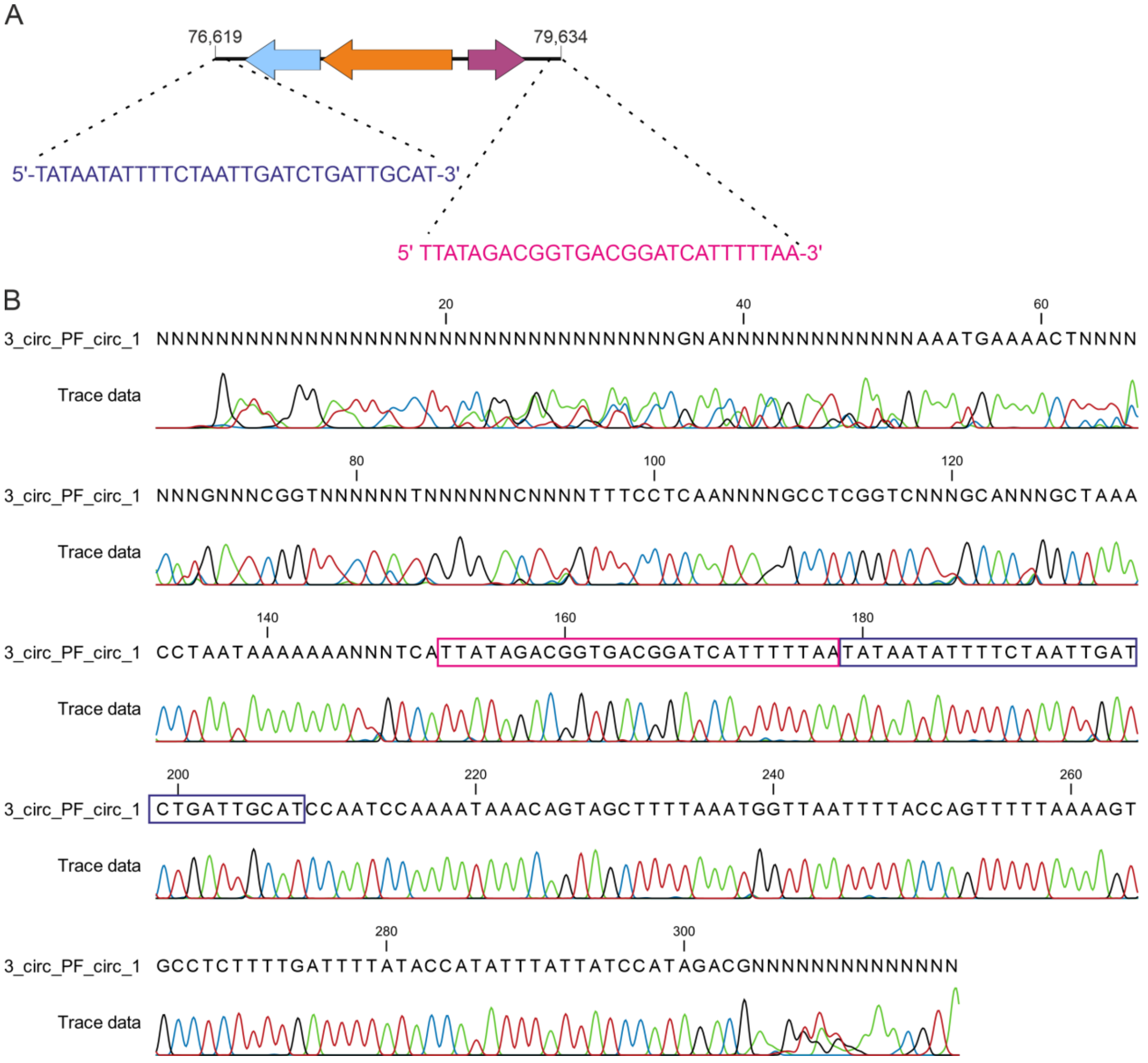

Figure S5. Detection of the circularized extrachromosomal element in *M. smithii* EVs. **A**, Schematic representation of the sequences at both termini of the element. **B**, Sequencing chromatogram confirming the results obtained by PCR (**Fig. 2C**).

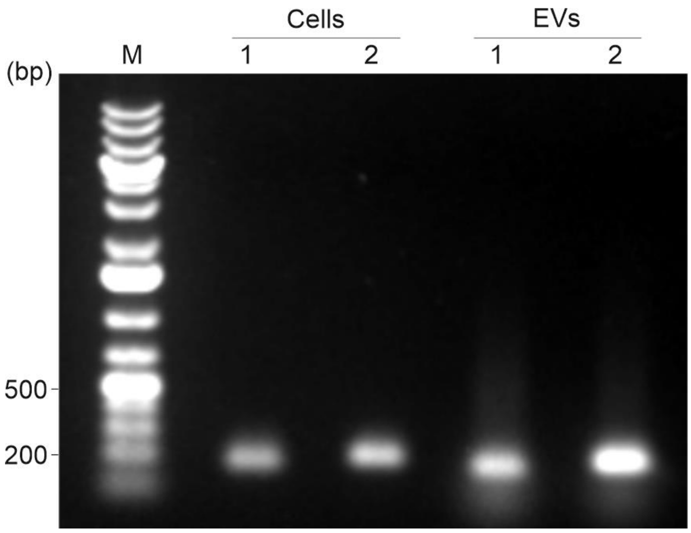

Figure S6. Detection of MSTV1 in both cells and purified EVs of *M. smithii* PS. The agarose gel electrophoresis shows the amplified products: lane 1, provirus integrated into the host chromosome (F: 5'-GGGTTTAATTTTGGGGGATA-3', R: 5'-AGGATTTCTTCATTGGTTCTCA-3'; expected size: 180 bp); lane 2, excised and circularized form of the MSTV1 genome (F: 5'TTGATGATGTTAATAATGGTGATGA-3', R: 5'-AGGATTTCTTCATTGGTTCTTCTCA-3'; expected size: 216 bp). M: Thermo Scientific GeneRuler 1 kb Plus DNA Ladder.

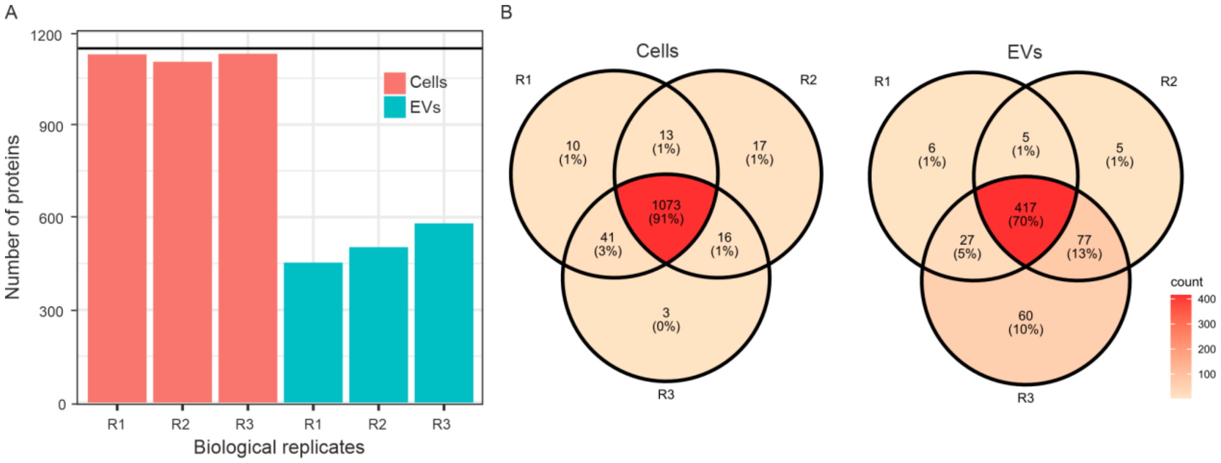

Figure S7. Proteomic analysis of *M. smithii* cells and EVs (n=3 biological replicates for EVs and *M. smithii* cells). **A**, Proteins identified per replicate for *M. smithii* cells and EVs. **B**, Venn diagram of the identified proteins per replicate for cells and EVs. The number and percentage reported in each circle indicate the total and relative amount of proteins found per sample.
