# Supplementary material for: Biogenesis of DNA-carrying extracellular vesicles by the dominant human gut methanogenic archaeon
Source: Nat Commun. 2025 Jun 3;16:5093. doi: 10.1038/s41467-025-60272-9 (PMC12134362; doi:10.1038/s41467-025-60272-9)
Supplement: Supplementary file 2 — Description of Additional Supplementary Files [file 41467_2025_60272_MOESM2_ESM.pdf]

Description of Additional Supplementary Files:

Supplementary Data 1: Proteins identified in the three replicates of cells and EVs samples (C=cells, V=vesicles).

Supplementary Data 2: Functional classification of arCOG categories.

Supplementary Data 3: Top 150 most abundant proteins in the *M. smithii* EVs based on the labelfree intensity-based absolute quantification (iBAQ).

Supplementary Data 4: Proteins identified in the three replicates of the cellular proteome.

Supplementary Data 5: Proteins enriched in *M. smithii* EVs after normalization with the cellular protein content. Differential protein abundances and adjusted p-values were calculated using the package DEP (Differential Enrichment analysis of Proteomics data) ($log2 > 1.5$, adj.p-value < 0.05). Source data are provided as a Source Data file.

Supplementary Movie 1: A tomogram of a cell with EVs trapped between the cytoplasmic membrane and peptidoglycan layer. The video of a tomogram and rendering corresponds to that in Figure 4A.

Supplementary Movie 2: EV release via blebbing/budding. The video of a tomogram and rendering corresponds to that in Figure 4A.
